# Effect of Proteinuria and Glomerular Filtration Rate on Renal Outcome in Patients with Biopsy-Proven Benign Nephrosclerosis

**DOI:** 10.1371/journal.pone.0147690

**Published:** 2016-01-25

**Authors:** Keiichi Sumida, Junichi Hoshino, Toshiharu Ueno, Koki Mise, Noriko Hayami, Tatsuya Suwabe, Masahiro Kawada, Aya Imafuku, Rikako Hiramatsu, Eiko Hasegawa, Masayuki Yamanouchi, Naoki Sawa, Takeshi Fujii, Kenichi Ohashi, Kenmei Takaichi, Yoshifumi Ubara

**Affiliations:** 1 Nephrology Center, Toranomon Hospital Kajigaya, Kanagawa, Japan; 2 Nephrology Center, Toranomon Hospital, Tokyo, Japan; 3 Department of Pathology, Toranomon Hospital, Tokyo, Japan; 4 Department of Pathology, Yokohama City University Graduate School of Medicine, Kanagawa, Japan; 5 Okinaka Memorial Institute for Medical Research, Toranomon Hospital, Tokyo, Japan; Mario Negri Institute for Pharmacological Research and Azienda Ospedaliera Ospedali Riuniti di Bergamo, ITALY

## Abstract

**Background:**

Reduced estimated glomerular filtration rate (eGFR) and proteinuria are risk factors for end-stage renal disease (ESRD), of which benign nephrosclerosis is a common cause. However, few biopsy-based studies have assessed these associations.

**Methods:**

We performed retrospective cohort study of 182 Japanese patients who underwent renal biopsy from June 1985 through March 2014 and who were diagnosed with benign nephrosclerosis. Competing risk regression analyses were used to investigate the effect of eGFR and proteinuria levels at the time of renal biopsy on the risk for renal events (ESRD or a 50% decline in eGFR from baseline).

**Results:**

During a median 5.8-year follow-up, 63 (34.6%) patients experienced renal events. The incidence of renal events increased with lower baseline eGFR and greater baseline proteinuria levels. After adjustment for baseline covariates, lower eGFR levels (subhazard ratios [SHRs], 1.30; 95% confidence interval [CI], 1.01–1.67, per 10 mL/min/1.73 m^2^) and higher proteinuria levels (SHR, 1.52; 95% CI, 1.23–1.87, per 1.0 g/day) at the time of renal biopsy were associated independently with higher risk for renal events. Lower levels of serum albumin (SHR, 2.07; 95% CI, 1.20–3.55 per 1.0 g/dL) were also associated with renal events. Patients with both eGFR <30 mL/min/1.73 m^2^ and proteinuria ≥0.5 g/day had a 26.7-fold higher risk (95% CI, 3.97–179.4) of renal events than patients with both eGFR ≥60 mL/min/1.73 m^2^ and proteinuria <0.5 g/day.

**Conclusions:**

Reduced eGFR and increased proteinuria as well as lower serum albumin at the time of renal biopsy are independent risk factors for renal events among patients with biopsy-proven benign nephrosclerosis.

## Introduction

Hypertensive nephrosclerosis is a common cause of end-stage renal disease (ESRD), accounting for 28.4% of incident cases of ESRD in the US, and for 12.3% in Japan [1,2]. The number of patients who develop ESRD from nephrosclerosis is increasing steadily [1,2].

The histopathologic features of benign nephrosclerosis include intimal thickening of arteries, arteriolar hyalinosis, or ischemic collapse of glomeruli [3,4], leading to glomerulosclerosis, interstitial fibrosis, and tubular atrophy. Hypertension is associated with these lesions, but it has also been suggested that other clinical factors such as aging, obesity, chronic inflammation, and oxidative stress might contribute to development of the pathological features of nephrosclerosis [5–8]. Furthermore, the diagnosis of nephrosclerosis is usually a presumption because histopathological confirmation is often lacking, especially among patients with hypertension [9]. These circumstances can engender inaccuracies of clinical diagnosis of benign nephrosclerosis and of risk prediction for clinical outcomes.

Reduced estimated glomerular filtration rate (eGFR) and increased proteinuria both have been demonstrated to be risk factors for progression to ESRD in patients with nephrosclerosis based on large clinical studies [10,11]. However, only a few cohort studies with small sample size have assessed the renal prognosis of patients with true benign nephrosclerosis that was confirmed by renal biopsy, much less the clinical impact of eGFR and proteinuria on renal outcomes [12,13]. In this regard, it is important to clarify the renal prognosis and the association of these two factors with renal outcome in a significant number of patients with biopsy-proven benign nephrosclerosis.

In the present study, we investigated renal prognosis and presented the clinical impact of baseline eGFR and proteinuria levels on the risk for renal events among patients with benign nephrosclerosis.

## Materials and Methods

### Study Population

**Fig 1:**
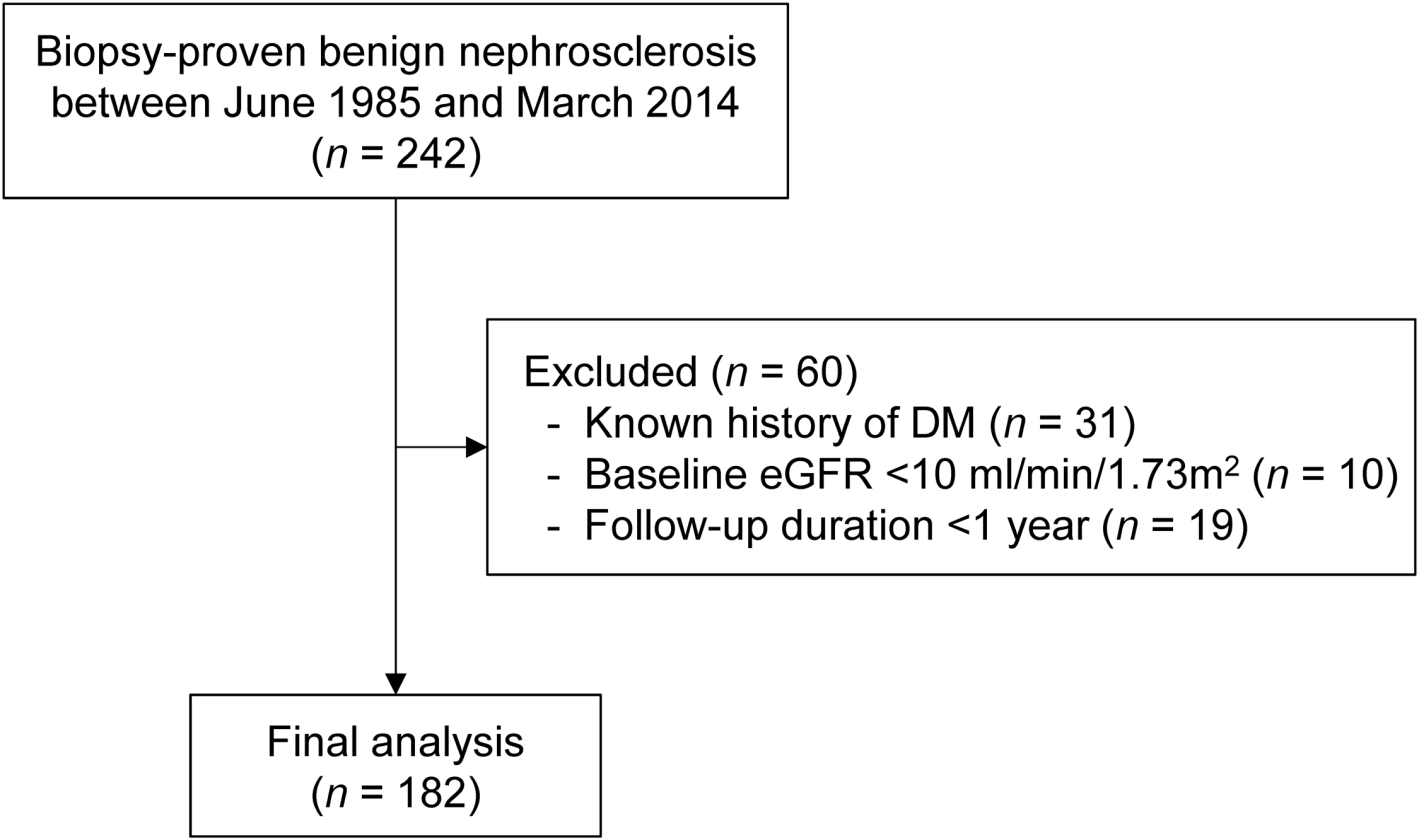
Flow chart of the study population. Abbreviations: DM, diabetes mellitus; eGFR, estimated glomerular filtration rate.

This retrospective cohort study examined patients with biopsy-proven benign nephrosclerosis who underwent native renal biopsies at Toranomon Hospital during June 1985 through March 2014. We reviewed the original renal biopsy reports and identified a total of 242 patients who had been confirmed to have benign nephrosclerosis by at least two renal pathologists and/or nephrologists at the time of renal biopsy. Excluded from those patients were those with a known history of diabetes, eGFR <10 mL/min/1.73 m^2^ at the time of renal biopsy, or with a duration of follow-up less than 1 year. No patient with accelerated or malignant hypertension was enrolled in this study. The final study population included 182 patients with biopsy-proven benign nephrosclerosis (Fig 1). This study was reviewed and approved with a waiver of the requirement to obtain informed consent received from the ethics committee of Toranomon Hospital. The patient records and information was anonymized and de-identified prior to analysis.

### Histopathological Diagnosis

All renal tissue specimens were obtained via percutaneous needle biopsies based on the decisions by our department and/or primary nephrologist. In general, standard indications for renal biopsy were unexplained proteinuria (≥0.5 g/day), hematuria (≥5 red blood cells/high power field), and/or decreased eGFR (<60 mL/min/1.73 m^2^). Standard procedures of processing and staining of biopsy specimens were used. All specimens were examined using light microscopy and immunohistochemistry including staining for IgG, IgA, IgM, C3, and C1q to exclude any immune deposits. Electron microscopy was also applied to exclude any immune deposits or renal disease of other types when considered necessary. The pathological diagnosis of benign nephrosclerosis was made based on typical histological features consistent with benign nephrosclerosis, including the presence of at least one of the following elements: intimal thickening of arteries, arteriolar hyalinosis, ischemic collapse or global sclerosis of glomeruli, or benign nephrosclerosis proportional to the extent of tubulointerstitial fibrosis in the absence of other defining lesions [3,4]. Cases with pathological findings of segmental glomerulosclerosis on kidney biopsy were included when primary focal segmental glomerulosclerosis (FSGS) was excluded according to the definition of primary FSGS in the Columbia classification system [14].

### Clinical and Laboratory Investigations

Baseline was defined as the time of renal biopsy. Age, sex, body mass index (BMI), BP, presence of hypertension, smoking history, pre-existing cardiovascular disease (CVD), and laboratory data including serum albumin, HbA1c, total cholesterol, serum uric acid, albumin-adjusted serum calcium [= serum calcium (mg/dL)—albumin (g/dL) + 4.0, if serum albumin <4.0 g/dL], serum phosphorus, C-reactive protein (CRP), hemoglobin, serum creatinine, eGFR, urine protein, and hematuria were obtained as clinical parameters at baseline. All laboratory values were measured using automated standardized methods at our hospital within 24 hr after drawing blood samples. Information related to the smoking history was obtained through a standard questionnaire. Hypertension was defined as blood pressure (BP) ≥140/90 mmHg using automatic devices in a sitting position, and/or use of antihypertensive agents. Pre-existing CVD included angina pectoris, myocardial infarction, cerebral hemorrhage, ischemic stroke, and atherosclerosis obliterans, which were obtained from medical records.

The eGFR (in mL/min/1.73 m^2^) was calculated using a formula for Japanese patients devised by Matsuo et al [15]. Baseline eGFR levels were treated as a continuous variable for primary analysis and also treated as categorical variables for a secondary analysis after dividing the eGFR levels into three categories based on the 2012 Kidney Disease: Improving Global Outcomes (KDIGO) classification of chronic kidney disease (CKD): ≥60 (CKD stage G1 or G2), 30 to <60 (CKD stage G3a or G3b), and <30 mL/min/1.73 m^2^ (CKD stage G4 or G5) [16]. Proteinuria at baseline was measured in a 24-hour urine sample; and was treated as a continuous variable for primary analysis and as categorical variables for a secondary analysis by classifying them as normal/mild proteinuria (<0.5 g/day) and moderate/severe proteinuria (≥0.5 g/day). Hematuria was defined as the presence of 5 or more red blood cells/high-power field. Average values during follow-up were also calculated for BP and urine protein. We extracted uses of angiotensin-converting enzyme inhibitors (ACEIs) or angiotensin receptor blockers (ARBs), calcium channel blockers (CCBs), and other antihypertensive agents (α-blockers, β-blockers, and diuretics), which were defined as administration for more than half of the follow-up period.

### Outcomes

The outcome for this study was defined as the first occurrence of renal events including ESRD requiring permanent dialysis or a 50% decline in eGFR from baseline. None of the study patients received kidney transplantation during the follow-up period. Patients were analyzed from the time of renal biopsy until renal events and were censored by death or the end of follow-up at March 31, 2015.

### Statistical Analysis

Data are presented as the number (percent) for categorical variables and the mean ± standard deviation for continuous variables with a normal distribution or median (IQR) for those with a skewed distribution. Categorical variables were analyzed with χ2 test. Continuous variables were compared using *t* tests, Mann–Whitney U tests, as appropriate. The distribution of the clinical parameters stratified by eGFR levels were compared using trend analysis. Survival curves were estimated using the Kaplan–Meier method and were compared by log-rank test. Patients lost to follow-up were regarded as censored at the date of the last documented contact in the following survival analyses.

To account for potential bias attributable to the competing risk of death before renal events, we performed univariate, age- and sex-adjusted, and multivariable-adjusted competing risk regression using a Fine–Gray competing-risks analysis to evaluate the association between potential covariates and renal events. In a multivariable model, the following important clinical variables were incorporated as covariates: baseline eGFR, urine protein, age, sex, BMI, systolic BP, smoking history, past history of CVD, serum albumin, serum uric acid, CRP, hemoglobin, and ACEIs/ARBs use. The joint association of baseline eGFR and proteinuria levels as categorical variables with the risk of renal events was also estimated for a secondary analysis. Patients with eGFR ≥60 mL/min/1.73 m^2^ and normal/mild proteinuria served as the reference group in the secondary analysis. We examined the possible interaction between baseline levels of eGFR and proteinuria on the risk for renal events by entering the interaction term in a multivariate competing risk regression model.

Of the variables included in a multivariable model, data points were missing for BMI (*n* = 5; 2.8%), serum uric acid (*n* = 3; 1.7%), CRP (*n* = 15; 8.2%), and hemoglobin (*n* = 3; 1.7%). Missing values were not imputed in the primary analysis, but were substituted after adding other clinically relevant variables (HbA1c [25.3% missing] and total cholesterol [24.2% missing]) with the use of multiple imputation procedures in a sensitivity analysis. Results are presented as subhazard ratios (SHRs) with 95% confidence intervals (CIs). For all analyses, *P* values less than 0.05 were inferred as significant. Analyses were conducted using software (Stata/IC ver. 12.1; StataCorp LP).

## Results

### Baseline Characteristics

Table 1 presents baseline characteristics of the study population stratified by eGFR and proteinuria levels at the time of renal biopsy. Overall, the mean age of patients was 55.8 ± 14.5 years; 62.6% were men. The median follow-up period was 5.8 years (IQR 2.7–10.3). Of the 182 patients, 58 (31.9%) had preserved renal function (eGFR ≥60 mL/min/1.73 m^2^), 84 (46.1%) with eGFR 30 to <60 mL/min/1.73 m^2^, and 40 (22.0%) with eGFR <30 mL/min/1.73 m^2^. The respective median follow-up periods were 6.5 (IQR 3.7–12.0), 6.6 (IQR 3.2–10.3), and 3.2 years (IQR 1.6–5.9) in patients with eGFR ≥60, 30 to <60, and <30 mL/min/1.73 m^2^.

**Table 1:** Baseline characteristics and average values during follow-up according to eGFR and proteinuria levels at the time of renal biopsy.

	All	eGFR at baseline (mL/min/1.73 m^2^)			*P*^a^	Proteinuria at baseline (g/day)		*P*	
Variable		≥60	30–<60	<30		Normal/mild (<0.5)	Moderate/severe (≥0.5)	
	(*N* = 182)	(*n* = 58)	(*n* = 84)	(*n* = 40)		(*n* = 85)	(*n* = 97)	
Age (years)	55.8±14.5	49.1±14.6	58.2±12.4	60.5±15.5	<0.001	51.9±13.6	59.3±14.5	<0.001
Male sex	114 (62.6)	40 (69.0)	53 (63.1)	21 (52.5)	0.25	51 (60.0)	63 (65.0)	0.49
Follow-up period (years)	5.8 [2.7, 10.3]	6.5 [3.7, 12.0]	6.6 [3.2, 10.3]	3.2 [1.6, 5.9]	0.001	7.6 [4.2, 12.7]	4.2 [1.9, 7.8]	<0.001
Body mass index (kg/m^2^)	24.0±3.9	25.4±4.6	23.6±3.3	23.1±3.6	0.002	24.1±4.2	23.9±3.6	0.35
Systolic BP (mmHg)	137.0±20.7	135.9±17.3	133.9±19.1	145.1±26.2	0.076	135.2±20.2	138.5±21.1	0.15
Diastolic BP (mmHg)	82.2±13.9	81.9±12.4	80.2±12.5	86.7±17.4	0.23	82.5±13.4	81.9±14.3	0.38
Hypertension	154 (84.6)	45 (77.6)	72 (85.7)	37 (92.5)	0.12	65 (76.5)	89 (91.8)	0.004
Smoking history	77 (42.3)	27 (46.6)	40 (47.6)	10 (25.0)	0.043	31 (36.5)	46 (47.4)	0.14
Pre-existing CVD	27 (14.8)	6 (10.3)	14 (16.7)	7 (17.5)	0.50	6 (7.1)	21 (21.6)	0.006
Laboratory parameters								
Serum albumin (g/dL)	3.7±0.6	3.9±0.6	3.7±0.6	3.4±0.7	<0.001	3.9±0.5	3.5±0.7	<0.001
HbA1c (%)	5.8±0.6	5.8±0.6	5.9±0.7	5.8±0.7	0.89	5.8±0.6	5.8±0.7	0.45
Total cholesterol (mg/dL)	198.8±40.2	194.8±37.1	199.5±39.5	202.9±46.0	0.48	195.9±38.3	201.4±42.0	0.21
Serum uric acid (mg/dL)	6.6±1.8	6.0±1.6	6.4±1.5	8.1±2.0	<0.001	6.2±1.7	7.0±1.8	0.002
Serum adjusted calcium (mg/dL)	9.3±0.6	9.3±0.4	9.3±0.8	9.4±0.6	0.80	9.3±0.7	9.3±0.5	0.19
Serum phosphorus (mg/dL)	3.5±0.7	3.4±0.6	3.4±0.5	3.7±0.9	0.062	3.6±0.6	3.4±0.7	0.036
C-Reactive protein (mg/dL)	0.1 [0, 0.4]	0.1 [0.1, 0.4]	0.1 [0, 0.4]	0.1 [0, 0.4]	0.96	0.1 [0, 0.3]	0.1 [0, 0.5]	0.26
Hemoglobin (g/dL)	13.1±2.4	14.3±1.9	13.4±2.2	11.0±2.2	<0.001	13.5±2.2	12.8±2.6	0.040
Serum creatinine (mg/dL)	1.4±0.8	0.8±0.2	1.2±0.3	2.6±1.0	<0.001	1.2±0.8	1.5±0.8	0.008
eGFR (mL/min/1.73 m^2^)	48.8±21.5	73.8±11.3	45.1±8.4	20.6±5.8	<0.001	54.8±21.0	43.6±20.8	<0.001
Urine protein (g/day)	0.5 [0.2, 1.3]	0.4 [0.2, 0.8]	0.6 [0.2, 1.3]	1.1 [0.4, 2.2]	<0.001	0.2 [0.1, 0.3]	1.2 [0.7, 2.0]	<0.001
Hematuria (≥5 /HPF)	37 (20.3)	12 (20.7)	20 (23.8)	5 (12.5)	0.34	18 (21.2)	19 (19.6)	0.79
Average values during follow-up								
Systolic BP (mmHg)	132.7±12.4	131.6±12.3	130.4±10.5	139.1±14.5	0.024	130.8±11.7	134.4±12.9	0.024
Diastolic BP (mmHg)	79.0±8.8	78.3±8.3	78.0±8.2	82.2±10.1	0.069	79.1±8.0	79.0±9.5	0.48
Urine protein (g/day)	0.7 [0.3, 1.7]	0.4 [0.2, 0.9]	1.0 [0.3, 2.1]	1.5 [0.5, 2.1]	<0.001	0.3 [0.1, 0.5]	1.0 [0.6, 1.3]	<0.001
ACEIs/ARBs use	91 (50.0)	26 (44.8)	39 (46.4)	26 (65.0)	0.098	33 (38.8)	58 (59.8)	0.005
CCBs use	83 (45.6)	23 (39.7)	35 (41.7)	25 (62.5)	0.051	31 (36.5)	52 (53.6)	0.021
Other antihypertensive agents use	54 (30.0)	11 (19.0)	30 (35.7)	13 (32.5)	0.09	23 (27.1)	31 (32.0)	0.47

*Note*: Values expressed as number (percentage), mean ± standard deviation, or median [interquartile range]. All laboratory parameters were obtained at baseline.

^a^ Tests for linear trend across eGFR categories.

Abbreviations: eGFR, estimated glomerular filtration rate; BP, blood pressure; CVD, cardiovascular disease; ACEIs, angiotensin-converting enzyme inhibitors; ARBs, angiotensin receptor blockers; CCBs, calcium channel blockers

Patients with lower eGFR levels were more likely to be older and hypertensive, and to have a higher BMI, higher levels of serum albumin and hemoglobin, and lower levels of serum uric acid and urine protein. Based on proteinuria, 97 patients (53.3%) had moderate/severe proteinuria at the time of renal biopsy. The distribution of patients according to the proteinuria levels is detailed in a histogram (S1 Fig). Those with moderate/severe proteinuria were more likely to be older and to have hypertension and history of CVD. They were more likely to have lower levels of serum albumin, serum phosphorus, and eGFR, and to have a higher level of serum uric acid than patients with normal/mild proteinuria.

### Clinical Parameters during Follow-Up

During follow-up, marked incremental trends of average systolic BP and urine protein were observed with decreasing eGFR levels (*P* for trend = 0.024 and <0.001, respectively). In patients with moderate/severe proteinuria, average systolic BP and the proportions of patients who used ACEIs/ARBs and CCBs were higher than those of patients with normal/mild proteinuria (Table 1).

### Incidence and Prognosis of Renal Events According to Baseline eGFR and Proteinuria Levels

Of the 182 patients, 63 (34.6%) experienced renal events during the follow-up period, including 10 patients (17.2%) with eGFR ≥60 mL/min/1.73 m^2^, 36 (42.9%) with eGFR 30 to <60 mL/min/1.73 m^2^, and 17 (42.5%) with eGFR <30 mL/min/1.73 m^2^. A total of 15 (8.2%) patients died during the follow-up period. Table 2 presents the number of patients and incidence rates of renal events stratified by eGFR and proteinuria levels. The incidence of renal events increased with lower baseline eGFR and greater baseline proteinuria levels, with the highest rate of incidence of 151.0 per 1000 person-years observed in patients with eGFR <30 mL/min/1.73 m^2^ and moderate/severe proteinuria (Table 2).

**Table 2:** Number of patients and incidence rates of renal outcome stratified by eGFR and proteinuria levels.

Proteinuria level at baseline (g/day)		eGFR level at baseline (mL/min/1.73 m^2^)		
		≥60	30 to <60	<30
		(*n* = 58)	(*n* = 84)	(*n* = 40)
Normal/mild proteinuria (<0.5)	(*n* = 85)	35 (9.1)	37 (27.9)	13 (55.5)
Moderate/severe proteinuria (≥0.5)	(*n* = 97)	23 (47.1)	47 (79.9)	27 (151.0)

*Note*: Values expressed as number (rate of incidence per person-years)

Abbreviations: eGFR, estimated glomerular filtration rate

**Fig 2:**
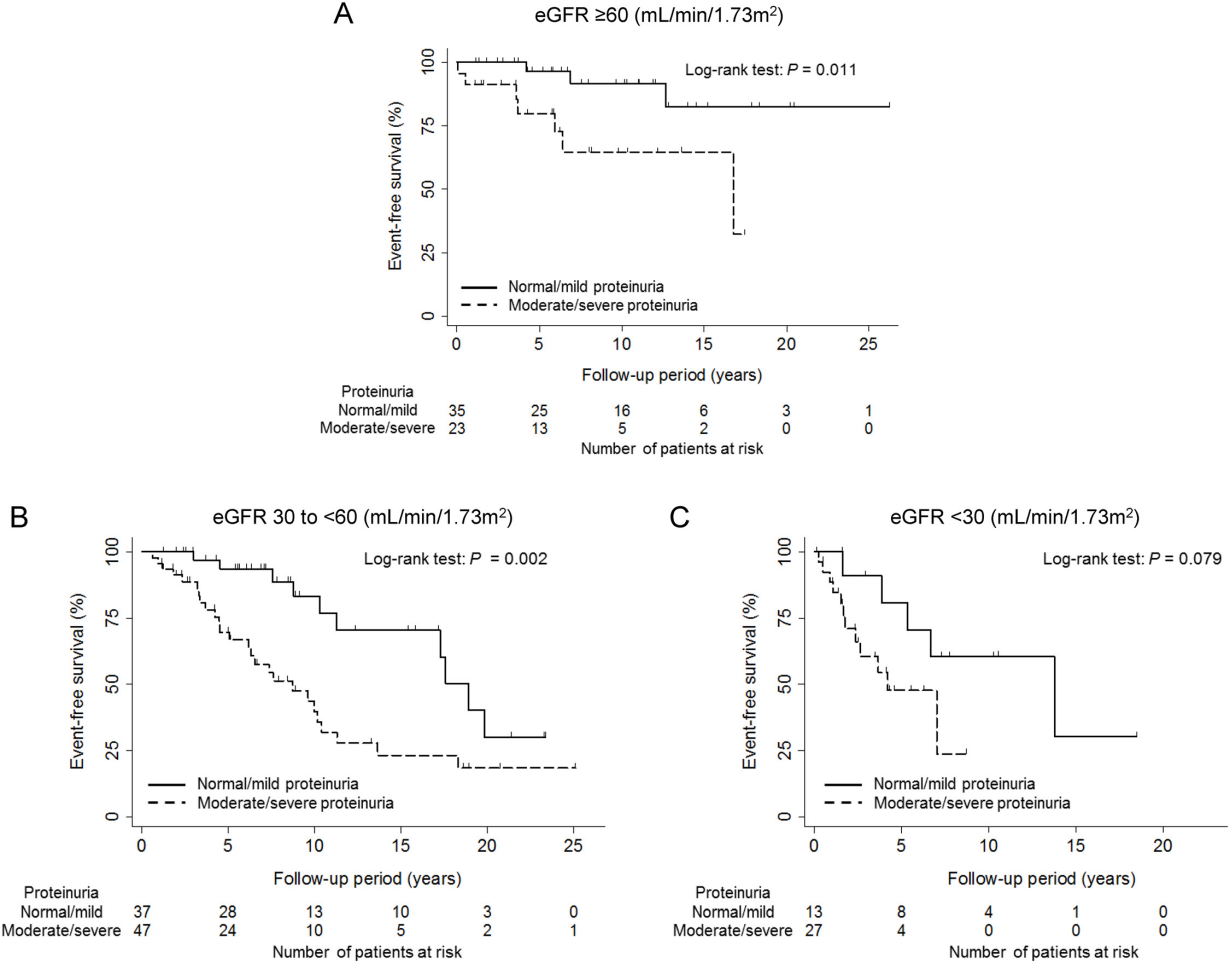
Event-free survival of renal events stratified by eGFR and proteinuria categories. A: eGFR ≥60 mL/min/1.73 m^2^. B: eGFR 30 to <60 mL/min/1.73 m^2^. C: eGFR <30 mL/min/1.73 m^2^. Abbreviation: eGFR, estimated glomerular filtration rate.

Event-free survival of renal events stratified by eGFR and proteinuria categories is shown in Fig 2. For both eGFR ≥60 and 30 to <60 mL/min/1.73 m^2^ categories, event-free survival was significantly lower in patients with moderate/severe proteinuria than in those with normal/mild proteinuria (*P* = 0.011 and 0.002, respectively, by the Log-rank test). Although the differences were not statistically significant, patients with moderate/severe proteinuria tended to have lower event-free survival than those with normal/mild proteinuria for eGFR <30 mL/min/1.73 m^2^ category (*P* = 0.079 by the Log-rank test).

### Risk Predictors for Renal Events

Risks for renal events were examined for three statistical models including pertinent clinical variables (Table 3). In unadjusted analyses, lower levels of eGFR and higher levels of proteinuria at baseline as well as higher age, smoking history, lower levels of serum albumin, and higher levels of serum uric acid were significantly associated with higher risk of renal events. After age- and sex-adjusted, and multivariable-adjusted analyses, both eGFR and proteinuria levels at baseline as well as serum albumin levels remained significant predictors of renal events. The multivariable-adjusted SHRs for renal events were 1.30 (95% CI, 1.01 to 1.67) for 10 mL/min/1.73 m^2^ decrease in eGFR levels and 1.52 (95% CI, 1.23–1.87) for 1.0 g/day increase in proteinuria levels (Table 3). No significant interaction was found between baseline eGFR and proteinuria levels (*P* >0.05 for interaction). Results of analyses in which imputed values for missing variables of HbA1c and total cholesterol were used yielded similar results (S1 Table).

**Table 3:** Association of clinical parameters with the risk for renal events.

	Unadjusted	*P*	Age- and sex-adjusted	*P*	Multivariable-adjusted	*P*
	SHR (95% CI)		SHR (95% CI)		SHR (95% CI)	
Baseline eGFR (per -10 mL/min/1.73 m^2^)	1.30 (1.13–1.50)	<0.001	1.29 (1.09–1.52)	0.003	1.30 (1.01–1.67)	0.041
Baseline proteinuria (per 1 g/day)	1.59 (1.33–1.91)	<0.001	1.66 (1.41–1.96)	<0.001	1.52 (1.23–1.87)	<0.001
Age (per 10 years)	1.24 (1.02–1.52)	0.030	1.27 (1.04–1.55)	0.017	0.96 (0.74–1.24)	0.76
Male sex	1.66 (0.96–2.88)	0.071	1.82 (1.02–3.26)	0.043	1.38 (0.65–2.95)	0.40
Body mass index (per 1 kg/m^2^)	0.95 (0.89–1.02)	0.16	0.94 (0.86–1.02)	0.14	0.90 (0.81–1.01)	0.061
Systolic BP (per 10 mmHg)	1.12 (0.97–1.30)	0.11	1.11 (0.95–1.28)	0.19	1.05 (0.90–1.23)	0.54
Smoking history	1.98 (1.21–3.24)	0.007	1.78 (0.96–3.31)	0.068	1.34 (0.64–2.83)	0.44
Pre-existing CVD	1.04 (0.51–2.11)	0.91	0.92 (0.45–1.89)	0.83	0.41 (0.14–1.17)	0.094
Serum albumin (per -1 g/dL)	1.93 (1.32–2.82)	0.001	1.87 (1.25–2.78)	0.002	2.07 (1.20–3.55)	0.009
Serum uric acid (per 1 mg/dL)	1.14 (1.01–1.29)	0.037	1.12 (0.99–1.27)	0.076	1.04 (0.84–1.30)	0.71
C-Reactive protein (per 1 mg/dL)	0.97 (0.87–1.09)	0.64	0.94 (0.81–1.08)	0.38	0.84 (0.63–1.12)	0.23
Hemoglobin (per 1 g/dL)	0.95 (0.85–1.05)	0.30	0.94 (0.84–1.06)	0.32	1.17 (0.99–1.38)	0.053
ACEIs/ARBs use	1.46 (0.90–2.36)	0.13	1.34 (0.81–2.21)	0.26	1.34 (0.75–2.38)	0.32

*Note*: Multivariate model is adjusted for baseline covariates, includes eGFR, proteinuria, age, sex, body mass index, systolic blood pressure, smoking history, past history of CVD, serum albumin, serum uric acid, C-reactive protein, hemoglobin, and ACEIs/ARBs use.

Abbreviations: eGFR, estimated glomerular filtration rate; SHR, subhazard ratio; CI, confidence interval; BP, blood pressure; CVD, cardiovascular disease; ACEIs, angiotensin-converting enzyme inhibitors; ARBs, angiotensin receptor blockers.

### Risk for Renal Events According to Baseline eGFR and Proteinuria Levels

**Fig 3:**
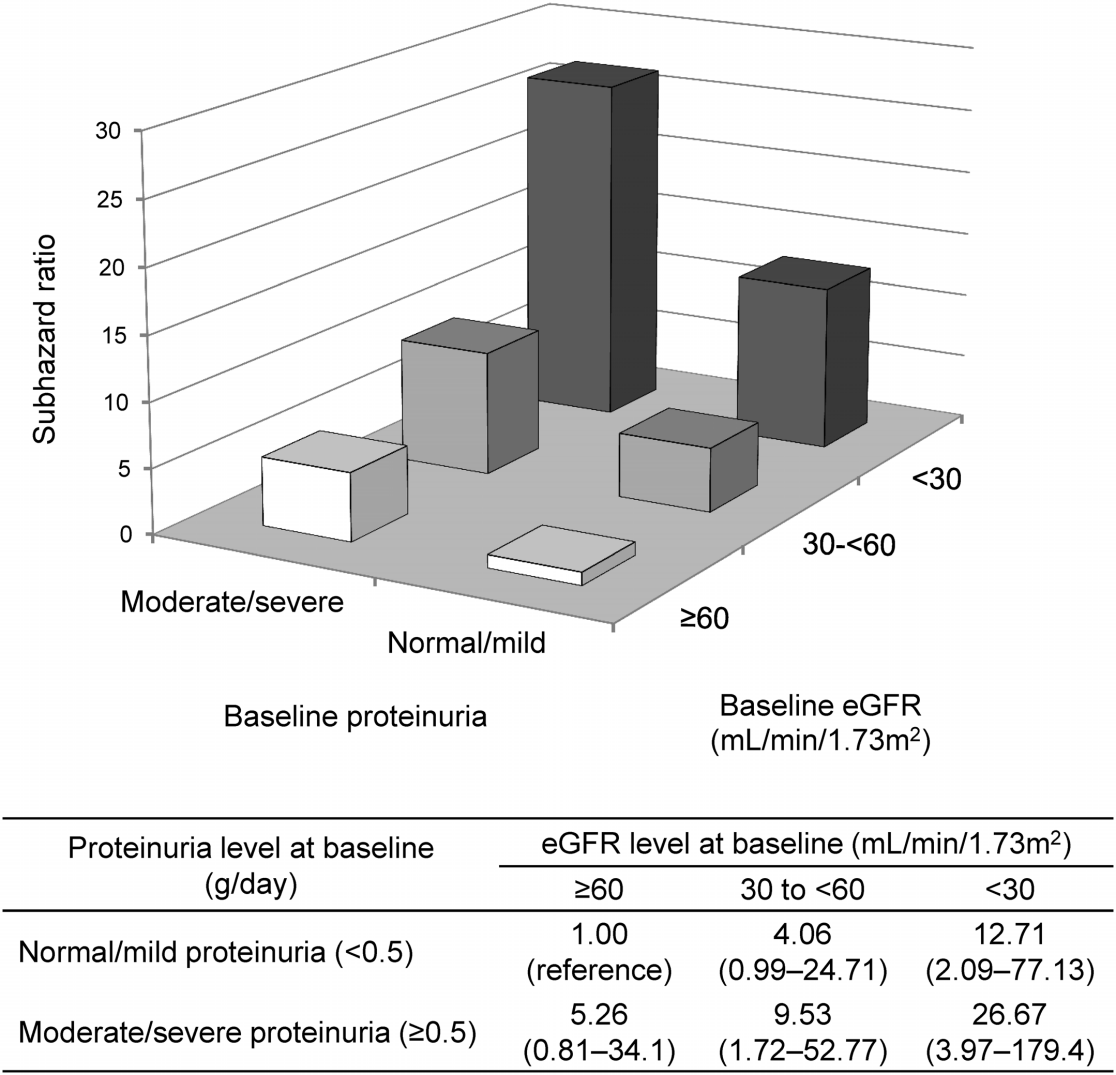
Joint association of baseline eGFR and proteinuria levels on the risk for renal events. Estimates are adjusted for baseline covariates, including age, sex, BMI, systolic blood pressure, smoking history, past history of CVD, serum albumin, serum uric acid, C-reactive protein, hemoglobin, and ACEIs/ARBs. The subgroup in the highest eGFR (≥60 mL/min/1.73 m^2^) and normal/mild proteinuria (<0.5 g/day) is the referent. Abbreviations: eGFR, estimated glomerular filtration rate; BMI, body mass index; CVD, cardiovascular disease; ACEIs, angiotensin-converting enzyme inhibitors; ARBs, angiotensin receptor blockers.

We also estimated the joint association of baseline eGFR and proteinuria levels as categorical variables with the risk for renal events (Fig 3). The effects of lower eGFR and higher proteinuria categories were independent of each other (*P* >0.05 for interaction). Multivariable-adjusted SHRs increased both with lower baseline eGFR and with greater baseline proteinuria levels. Compared to patients with eGFR ≥60 mL/min/1.73 m^2^ and normal/mild proteinuria, those with both eGFR <30 mL/min/1.73 m^2^ and moderate/severe proteinuria had a 26.7-fold higher risk (95% CI 3.97 to 179.4) of renal events.

## Discussion

This retrospective cohort study examined clinical features with accompanying long-term renal outcome in patients with biopsy-proven benign nephrosclerosis and demonstrated the clinical impact of eGFR and proteinuria on renal outcome among these patients. Results show that baseline reduced eGFR and increased proteinuria as well as lower serum albumin are associated independently with the risk for renal events, which increased significantly with decrement in eGFR particularly under the presence of moderate/severe proteinuria.

Several clinical factors have been suggested as risk factors influencing in the development of nephrosclerosis, including age, African descent, systolic BP level, proteinuria, and concomitant CVD [9]. However, promising risk factors for unfavorable renal outcomes have not been fully identified among patients with definite diagnosis of benign nephrosclerosis because the diagnosis is usually a presumption with no histopathologic confirmation. To date, a few biopsy-based studies have found clinical and pathological findings as independent risk factors for poor renal prognosis. These factors include reduced renal function, proteinuria, elevated BP, and a severe grade of global glomerulosclerosis [12,13,17,18].

Vikse et al. reported that serum creatinine and proteinuria at the time of renal biopsy were significant predictors of progression of ESRD among 102 patients with biopsy-proven nephrosclerosis during a median follow-up of 11.7 years [12]. Dasgupta et al. examined 60 patients with hypertensive benign nephrosclerosis diagnosed on renal biopsy for a mean follow-up of 6.7 years. Their results revealed that only mean diastolic BP during follow-up predicted renal survival, with 5- and 10-year renal survival results of 56% and 35%, respectively [18]. Recently, Suzuki et al. assessed 35 Japanese patients with biopsy-proven benign nephrosclerosis during a 10 year follow-up period, and concluded that eGFR at the time of renal biopsy was the strongest risk factor for composite outcomes of ESRD, CVD, and death [13].

In earlier biopsy-based studies, however, treatment effect of ACEIs/ARBs against angiotensin II, which has been regarded as playing a major role in the progression of nephrosclerosis [16], was not examined through risk analysis of renal outcomes. The present study analyzed the risk for renal outcome using multivariable adjustment including the use of ACEIs/ARBs during follow-up as well as other clinically relevant factors. This study identified the baseline eGFR and proteinuria as strong prognostic factors for poor renal outcome, which is consistent with results presented in previous reports. Furthermore, we demonstrated the joint association of baseline eGFR and proteinuria levels on the risk for renal events. Our results, however, revealed no significant association between baseline BP and renal events from either univariate or multivariate analysis.

Hypertension causes renal vascular lesion leading to glomerular and tubulointerstitial injury. Moreover, clinical factors including aging and metabolic derangements such as obesity, chronic inflammation, and oxidative stress may also contribute to the development of the renal lesions of nephrosclerosis [5–8]. These results indicate that hypertension is not necessarily a pre-requisite for these lesions to occur [19]. In fact, various studies of human and animal experimental models have demonstrated that renal vessel changes may precede the onset of hypertension or appear in the absence of hypertension [20–22]. It is particularly interesting that the present study also included 28 of 182 patients (15.2%) of nephrosclerosis without apparent history of hypertension. Although we were unable to detect any specific clinical features among those patients, distinct pathophysiologic mechanisms or clinical characteristics of these patients might underlie the development of nephrosclerosis without hypertension.

Regarding the pathogenesis of benign nephrosclerosis, two major pathophysiologic mechanisms have been suggested to induce its glomerular lesions and tubulointerstitial fibrosis: loss of autoregulation of the renal blood flow caused by arteriolohyalinosis [23,24], and ischemia that fosters the generation of hypoxia-inducible fibrosing factors [25–28]. Arteriolosclerosis and arteriolohyalinosis play a major role in inducing glomerular ischemic shrinking and sclerosis along with glomerulomegary and FSGS [19]. These lesions can be accompanied by tubulointerstitial inflammation and fibrosis through the pathophysiologic mechanisms above, and can engender the decline of renal function [19]. Although proteinuria has long been regarded as a reflection of the extent and severity of glomerular damage, it is a key aspect of the pathogenesis of progressive renal dysfunction. Protein-tubular cell interactions have inflammatory and fibrogenic consequences that can contribute to the main feature of nephrosclerosis; interstitial damage and fibrosis [29,30]. Taken together, it is likely that both reduced eGFR and proteinuria are important markers for the development of renal dysfunction in patients with benign nephrosclerosis. In the present study, serum albumin was also identified as a prognostic factor for renal events independent of other clinical factors including both baseline eGFR and proteinuria. Although the precise mechanisms underlying the increased renal events seen in patients with lower levels of serum albumin remain speculative, chronic malnutrition and/or inflammation status, frailty, or decreased plasma oncotic pressure could potentially contribute to the progression of benign nephrosclerosis.

From several clinical studies, comparable findings have also been reported for the baseline eGFR and proteinuria as clinical prognostic factors for renal outcomes. Community-based cohort study from the Alberta Kidney Disease Network including 920,985 adult participants reported that the presence of heavy proteinuria and reduced eGFR were associated with higher risk for progression to kidney failure [10]. Results from the African American Study of Hypertension and Kidney Disease of 1094 black individuals with hypertensive nephrosclerosis also demonstrated that baseline GFR and proteinuria were highly predictive of an increased risk for composite clinical renal outcomes of 50% or 25-ml/min/1.73 m^2^ GFR decline or ESRD [11]. These larger cohort studies were limited, however, because of a lack of histopathological confirmation prevented assessment of the diagnosis of underlying renal disease. Therefore, in addition to these studies, our biopsy-based study highlights the clinical value of eGFR and proteinuria for risk assessment related to renal prognosis for individuals with benign nephrosclerosis who might benefit from preventive measures.

Results of this study must be interpreted in light of several limitations. First, this is a small, single-center retrospective cohort study with a restricted population of patients who underwent renal biopsy, which might present limitations to the statistical power and generalizability of our results, perhaps rendering them less applicable to other populations with benign nephrosclerosis. Second, the indications for renal biopsy were not fully standardized, making it undeniable that there was selection bias in our study. Third, follow-up data of clinical factors that might have affected renal outcome were not assessed using time-weighted average in the regression analyses. However, preliminary multivariable-adjusted analysis, including a time-averaged systolic BP as a covariate which can have a strong influence on renal events, also retained baseline eGFR and proteinuria as significant risk factors for the outcome. Finally, pathological findings such as glomerular sclerosis, arterial or arteriolar lesions, interstitial fibrosis, and tubular atrophy were not included as covariates in this study. Nonetheless, our results are important, presenting novel findings that strengthen the clinical significance of eGFR and proteinuria for renal outcomes in patients with biopsy-proven benign nephrosclerosis.

In conclusion, our results demonstrate that reduced eGFR and increased proteinuria as well as lower serum albumin at the time of renal biopsy have significant effects as strong risk factors for long-term renal outcome among patients with benign nephrosclerosis. Further studies including pathological findings are necessary to identify additional relevant prognostic factors in patients with benign nephrosclerosis.

## Supporting Information

S1 Fig The histogram of patients according to six categories of proteinuria levels at baseline. (PDF)

S1 Table Multivariate-adjusted subhazard ratios for renal events after additional adjustment for laboratory data with imputation. (PDF)
