# Supplementary figures and images for: Effect of Proteinuria and Glomerular Filtration Rate on Renal Outcome in Patients with Biopsy-Proven Benign Nephrosclerosis
Source: PLoS One. 2016 Jan 25;11(1):e0147690. doi: 10.1371/journal.pone.0147690 (PMC4726632; doi:10.1371/journal.pone.0147690)

S1 Fig

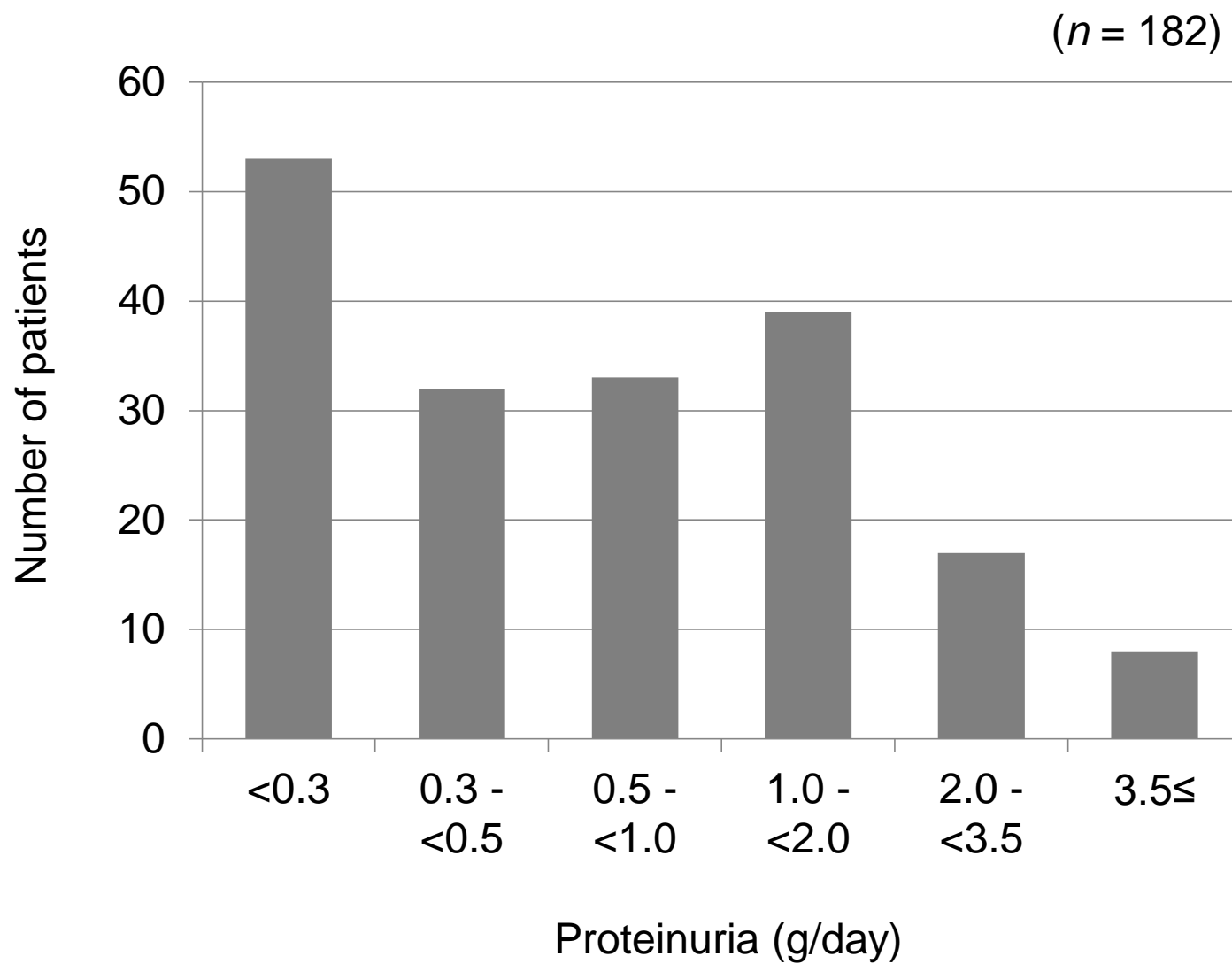

Supplement: S1 Fig — (PDF) [file pone.0147690.s001.pdf]
