# Supplementary material for: Effect of Proteinuria and Glomerular Filtration Rate on Renal Outcome in Patients with Biopsy-Proven Benign Nephrosclerosis
Source: PLoS One. 2016 Jan 25;11(1):e0147690. doi: 10.1371/journal.pone.0147690 (PMC4726632; doi:10.1371/journal.pone.0147690)
Supplement: S1 Table — (PDF) [file pone.0147690.s002.pdf]

S1 Table. Multivariate-adjusted subhazard ratios for renal events after additional adjustment for laboratory data with imputation.

	Multivariable-Adjusted	*P*
	SHR (95% CI)	
Baseline eGFR (per -10 mL/min/1.73 m^2)	1.30 (1.02–1.66)	0.033
Baseline proteinuria (per 1 g/day)	1.50 (1.21–1.87)	<0.001
Age (per 10 years)	0.95 (0.73–1.23)	0.69
Male sex	1.58 (0.64–3.92)	0.32
Body mass index (per 1 kg/m^2)	0.89 (0.80–1.00)	0.055
Systolic BP (per 10 mmHg)	1.03 (0.88–1.21)	0.67
Smoking	1.48 (0.66–3.32)	0.34
Pre-existing CVD	0.40 (0.13–1.20)	0.10
Serum albumin (per -1 g/dL)	2.10 (1.22–3.61)	0.007
Serum uric acid (per 1 mg/dL)	1.04 (0.83–1.30)	0.73
C-Reactive protein (per 1 mg/dL)	0.84 (0.62–1.15)	0.28
Hemoglobin (per 1 g/dL)	1.16 (0.99–1.36)	0.075
HbA1c (per 1 %)	0.86 (0.57–1.30)	0.47
Total cholesterol (per 10 mg/dL)	1.04 (0.93–1.15)	0.37
ACEIs/ARBs use	1.29 (0.72–2.30)	0.37

Note: Data were adjusted for baseline covariates, includes eGFR, proteinuria, age, sex, body mass index, systolic blood pressure, smoking history, past history of CVD, serum albumin, serum uric acid, C-reactive protein, hemoglobin, HbA1c, total cholesterol, and ACEIs/ARBs use.

Abbreviations: eGFR, estimated glomerular filtration rate; SHR, subhazard ratio; CI, confidence interval; BP, blood pressure; CVD, cardiovascular disease; ACEIs, angiotensin-converting enzyme inhibitors; ARBs, angiotensin receptor blockers.
